# Glaucoma Clinical Research: Trends in Treatment Strategies and Drug Development

**DOI:** 10.3389/fmed.2021.733080

**Published:** 2021-09-13

**Authors:** Line Storgaard, Thuy Linh Tran, Josefine Clement Freiberg, Alexander S. Hauser, Miriam Kolko

**Affiliations:** ^1^Department of Drug Design and Pharmacology, University of Copenhagen, Copenhagen, Denmark; ^2^Department of Ophthalmology, Copenhagen University Hospital, Rigshospitalet-Glostrup, Copenhagen, Denmark

**Keywords:** glaucoma, clinical trials, trends, treatment, drug development

## Abstract

**Purpose:** To investigate the trends and progresses in glaucoma research by searching two major clinical trial registries; clinicaltrials.gov, and Australianclinicaltrials.gov.au.

**Methods:** All clinical trials with glaucoma covered by Clinicaltrials.gov, and Australianclinicaltrials.gov.au starting the study before 1 January 2021 were included. Trials evaluating glaucoma treatment were separated from non-treatment trials and divided into three major categories: “laser treatment,” “surgical treatment,” and “medical treatment.” In the category of “medical treatment,” new compounds and their individual targets were identified and subcategorized according to treatment strategy; intraocular pressure (IOP)-lowering, neuroprotective or vascular. The phase transition success rates were calculated.

**Results:** One-thousand five hundred and thirty-seven trials were identified. Sixty-three percent (*n* = 971) evaluated glaucoma treatment, of which medical treatment accounted for the largest proportion (53%). The majority of medical trials evaluated IOP-lowering compounds, while trials with neuroprotective or vascular compounds accounted for only 5 and 3%, respectively. Eighty-eight new compounds were identified. Phase I, II, and III transition success rates were 63, 26, and 47%, respectively.

**Conclusion:** The number of clinical trials in glaucoma research has increased significantly over the last 30 years. Among the most recently evaluated compounds, all three main treatment strategies were represented, but clinical trials in neuroprotection and vascular modalities are still sparse. In addition to traditional medicines, dietary supplements and growth factors are assessed for a potential anti-glaucomatous effect. Phase II and III success rates were below previously reported success rates for all diseases and ophthalmology in general. A stricter phenotyping of patients can improve the success rates in glaucoma and ophthalmological research and gain a better understanding of responders and non-responders.

## Introduction

Glaucoma is one of the leading causes of global irreversible blindness, and the prevalence is increasing (1, 2). Glaucoma is characterized by a progressive degeneration of the optic nerve with corresponding visual field loss and ultimately blindness if left untreated. The pathophysiology of glaucoma is multifactorial and there are several clinical phenotypes (3). In short, glaucoma can be divided into primary glaucoma, secondary glaucoma and the rarer forms of juvenile and congenital glaucoma. Within primary glaucoma, there are two clinical phenotypes, open-angle glaucoma (POAG) and angle-closure glaucoma. In all glaucoma subtypes, elevated intraocular pressure (IOP) is recognized as a major risk factor for the development and progression of glaucoma and lowering IOP is currently the only documented method of treating glaucoma.

Numerous trials are being conducted around the world to examine glaucoma with the aim of improving glaucoma diagnosis, management, and treatment of the disease. Among these, clinical trials evaluating either medical, surgical, or behavioral intervention are of great interest. Clinical trials advance through four phases to test a particular treatment, find an appropriate dose of a given drug, and evaluate side effects of the treatment. When U. S. Food and Drug Administration (FDA) or a similar ruling authority decide whether a drug should be approved, Phase I, II, and III trials are usually conducted in advance. Phase IV trials are post-marketing or surveillance studies performed to monitor adverse reactions, safety, long-term risks, benefits and efficacy in large and diverse populations over several years (4).

The first interventional glaucoma trial dates back to 1978, according to the database clinicaltrials.gov, whereas the first article listed in PubMed based on a clinical trial was published in 1961 (5). Various aspects of glaucoma are evaluated in clinical trials, including diagnostic tests, procedures, devices, drugs, and behavioral factors. This paper presents data on all clinical trials of glaucoma covered by the two major trials registers clinicaltrials.gov and australianclinicaltrials.gov.au. ClinicalTrials.gov is a database provided by the U.S. national Library of Medicine at the National Institutes of Health. It provides information on both privately and publicly funded clinical studies and provides access to summary information and trial results on a wide range of diseases and conditions (6). australianclinicaltrials.gov.au is a joint initiative between the National Health and Medical Research Council and the Department of Industry, Innovation and Science in Australia (7). The Australian registry was included to ensure that clinical trials conducted at Melbourne Centre for Eye Research Australia would be covered by this review. We were aware that important trials evaluating the role of vitamin B3 supplementation in treating glaucoma did not appear in the registry clinicaltrials.gov.

All new compounds evaluated for the treatment of glaucoma and their individual targets are presented with the aim of showing the trends in drug development over the last decades. The objective of this paper is to provide an overview of the different types of glaucoma treatments and to investigate whether the strategies and targets in glaucoma drug development have changed over time. Furthermore, this paper examines how the different compounds perform in progressing through the different phases of clinical trials. Additional, personalized medicine and mathematical modeling are discussed as potential strategies to improve the possibility of successful glaucoma therapy in the future.

### Treatment of Glaucoma

Glaucoma treatment falls into three basic categories: laser treatment, incisional surgery, and medication (8, 9).

Laser treatment can be used in several ways to treat glaucoma: targeting the trabecular meshwork and thereby reducing IOP; performing peripheral laser iridotomy to prevent pupillary block; ablating the ciliary processes to reduce production of aqueous humor; and facilitating surgical procedures, such as trabeculectomy (10, 11).

Argon laser trabeculoplasty (ALT) was introduced as a treatment modality by Wise and Witter in 1979 (12), and ~20 years later in 1998, selective laser trabeculoplasty (SLT) was introduced by Latina et al. SLT is currently the most frequently used and accepted laser therapy in the treatment of POAG (13). Most clinical trials regarding glaucoma laser treatment registered in clinicaltrials.gov evaluate traditional laser modalities, whereas more recently micropulse laser trabeculoplasty and micropulse transscleral cyclophotocoagulation have been evaluated in clinical trials (14, 15).

Surgery is typically performed when non-invasive efforts (maximal tolerated medical therapy and/or laser trabeculoplasty) have not reached target IOP levels. Trabeculectomy is currently the most frequently performed glaucoma filtration procedure. Recently, less invasive glaucoma procedures, such as minimally invasive glaucoma surgery (MIGS), have gained popularity with new devices entering the market on a regular basis (13, 16). Recent trials have focused on examining the outcomes of conventional glaucoma surgery, including trabeculectomy and glaucoma drainage devices. In addition, the use of minimally invasive devices for glaucoma surgery devices has been investigated.

Medical treatment (e.g., topical eye drops) is considered a reasonable first choice of therapy in published guidelines for the treatment of POAG (17, 18). Clinicians usually prescribe a single drug selected from one of four drug classes—prostaglandin analogs (PGAs), beta-blockers, carbonic anhydrase inhibitors, and alpha-2 adrenergic agonists. Furthermore, miotic drugs can be an alternative, but are now almost never used as a first-line treatment due to side effects (19).

### History of Glaucoma Pharmacology

The history of glaucoma pharmacology began almost 150 years ago. Cholinergic drugs, also known as parasympathomimetics or miotics, were the first class of drugs used to treat glaucoma. Eserine (physostigmine) was the first glaucoma drug, a cholinergic agonist from 1876. Pilocarpine, the second miotic, was launched just a year later and showed fewer adverse events and was thus better tolerated by patients (20). Osmotic agents were added to the list of available agents in the early 1900s (21). The second class of IOP-lowering drugs, the adrenergic agonist, debuted with epinephrine in 1901. Epinephrine first became commercially available for glaucoma in the 1950s, followed shortly after by clonidine (20). Systemic carbonic anhydrase inhibitors were introduced in 1954 (22), but patients experienced a number of side effects. Topical formulations of the systemic carbonic anhydrase inhibitors were attempted to be prepared, but the formulations at that time had little or no effect on IOP (20).

In the early 1960s, propranolol was discovered and became the first commercially successful beta-blocker (23). The IOP-lowering effects of beta-adrenergic antagonists were discovered in 1967. However, the drug was not available as a topical agent due to corneal anesthetic properties and a negative effect on tear production (20). A decade later, timolol became available in 1978 (24). For a while, topical beta-blockers became the most prescribed anti-glaucomatous treatment until prostaglandin analogs were introduced on the market. The alpha-adrenergic agonist apraclonidine, a derivative of clonidine that is highly selective for the alpha2 receptor, was introduced around 1987. In 1996, brimonidine reached the market and largely replaced apraclonidine as the preferred adrenergic agonist for glaucoma.

In 1995, after many years of research, dorzolamide, a topical carbonic anhydrase inhibitor, finally reached the market following FDA approval. Today, dorzolamide is still widely used in the clinic (25). In the mid-1990s, prostaglandin analogs revolutionized the medical treatment of glaucoma (26, 27). Latanoprost was the first prostaglandin analog to receive FDA approval in 1996. Hereafter, bimatoprost, travoprost, tafluprost and the partial agonist unoprotone were subsequently approved (27). In 2017, a nitric oxide-donating prostaglandin analog, latanoprostene bunod (27) was approved by FDA.

Preservatives used in topical glaucoma medications may have toxic effects on the ocular surface, especially in patients receiving a multiple drop regimen (28, 29). In particular, the adverse effects of by far the most common preservative benzalkonium chloride (BAK) are well-described (30, 31). In recent decades, several glaucoma eye drops have been reformulated into preservative-free versions, and some have changed preservatives. A challenge of preservative-free formulations is the absence of an antimicrobial effect and thus an increased risk of contamination (28). Single-dose units are therefore frequently used as alternative but are more expensive and can be difficult to handle. Newer multi-dose formulations have been developed which dispense droplets either by a non-return valve or a filtration systems to ensure sterility after opening (28).

A new class of glaucoma drugs and the first major innovation in glaucoma therapy after 2000s were signaled by the approval of the first Rho kinase inhibitors, ripasudil in Japan in 2014 followed by netarsudil in the U.S in 2017 (32) and by European Medicine Agency (EMA) in 2019. The combination therapy netarsudil/latanoprost was approved by the FDA in 2019 and by the EMA in January 2021. Currently, the combination of ripasudil with the alpha2 agonist brimonidine (started Phase III in Japan February 2020) and the combination of ripasudil (or netarsudil) with sepetaprost (32) are in the pipeline.

### Neuroprotection in Glaucoma

Current available therapies for glaucoma have the primary aim of reducing IOP without directly addressing the associated optic neuropathy and retinal ganglion cell loss (33, 34).

Although lowering IOP is the primary treatment target in glaucoma management, there is a growing interest in neuroprotective strategies, as reducing IOP is often not sufficient to slow disease progression.

Neuroprotection in glaucoma refers to non-IOP-related interventions that prevent or delay glaucomatous neurodegeneration independent of IOP. Neuroprotection for glaucoma has been demonstrated in several animal models (35). So far, however, no relevant effect has been demonstrated in clinical trials in humans. Memantine, an N-methyl-d-aspartate antagonist, has been shown to be effective in neurodegenerative disorders but has no effect in glaucoma. A comprehensive phase 3 randomized multicenter clinical trial lasting more than 5 years at significant costs did not reveal benefits for memantine treatment by preventing the progression of visual field loss in glaucoma patients (36). Brimonidine, an α2-adrenoreceptor agonist widely distributed in the retina and anterior segment of the eye, may slow visual field deterioration, but a randomized controlled trial comparing 0.2% brimonidine with 0.5% timolol did not provide convincing evidence (37).

### Ocular Blood Flow in Glaucoma

It has been suggested that changes in ocular blood flow may alter the retinal functions affecting the prognosis for glaucoma. Several studies have shown a reduction of ocular blood supply in patients with both preperimetric as well as advanced glaucoma, suggesting an association between reduction in blood flow and glaucomatous damage (38–40). Abnormalities in retinal blood flow may play a role in the etiology of glaucoma, but the underlying pathophysiological mechanisms are still uncertain. Furthermore, only a limited proportion of patients with vascular deficiency develop glaucoma, and therefore vascular dysregulation is thought to be only one of many risk factors for developing glaucoma (41).

### Dietary Supplements

A number of trials are investigating the potential beneficial anti-glaucomatous effects of dietary supplements. Dietary supplements include vitamins, minerals, herbs, amino acids, and enzymes. Dietary supplements are not intended for the treatment, diagnosis, prevention or cure of diseases (42). Unlike pharmaceutical products, which must be proven safe and effective by regulatory authorities before marketing, manufacturers, and distributors of dietary supplements are solely responsible for ensuring that their products are safe before entering the market.

## Methods

An in-dept query on Clinicaltrials.gov and Australianclinicaltrials.gov.au was performed using the keyword “glaucoma” and all registered trials were included. We did not distinguish between investigational and observational trials. Data were extracted January 2021. All trials were sorted by start date of the study and grouped into time intervals: before 2000, 2000–2004, 2005–2009, 2010–2014, 2015–2019, and 2020. Based on the details of the study record, we separated studies regarding treatment of glaucoma from non-treatment studies. We included studies evaluating medical treatment aimed at other diseases than glaucoma, but with a possible secondary effect on IOP. These studies were not included in the figures, but the numbers are shown in the tables.

The non-treatment group included studies that assessed quality of life, adherence, diagnostic methods, and epidemiology.

The treatment group was further sub-divided into laser treatment, surgical treatment, or medical treatment. For all trials evaluating medical treatment, we identified the specific compound being evaluated. We distinguished between compounds already approved for the treatment of glaucoma and new compounds, including repurposed drug candidates. New compounds were subcategorized according to treatment strategy; IOP-lowering, neuroprotective, or agents that act on microvascular blood flow. Trials characterized as vascular research included studies that analyzed the following parameters: retinal or choroidal blood flow, optic disc blood flow, or retrobulbar vascular resistance.

We recorded recruitment status and phase for each trial. Target information for all new compounds was collected from the online resources Drugbank, Open Targets and from the literature (43, 44). A new compound was defined successful if it went from one phase to the next. Trials registered with the following recruitment status were defined as ongoing: “Not yet recruiting,” “recruiting,” “enrolling by invitation,” “active, not recruiting,” and “unknown.” New compounds were defined as treatment with the indication of glaucoma approved after 2015. Ongoing trials and trials completed within 2 years were considered potentially successful and categorized as such. However, compounds evaluated in ongoing trials with a start date of the study more than 4 years ago were marked as unsuccessful in the success calculation due to an expected duration of maximum 4 years of a clinical trial (45). Success rates were calculated per phase. By calculating the number of compounds that advance to the next phase vs. the total number of compounds per phase, we assessed the success rate at each of the three development phases. The phase transition success rates per phase were compared with previously reported success rates for clinical drug development (46).

## Results

**Figure 1:**
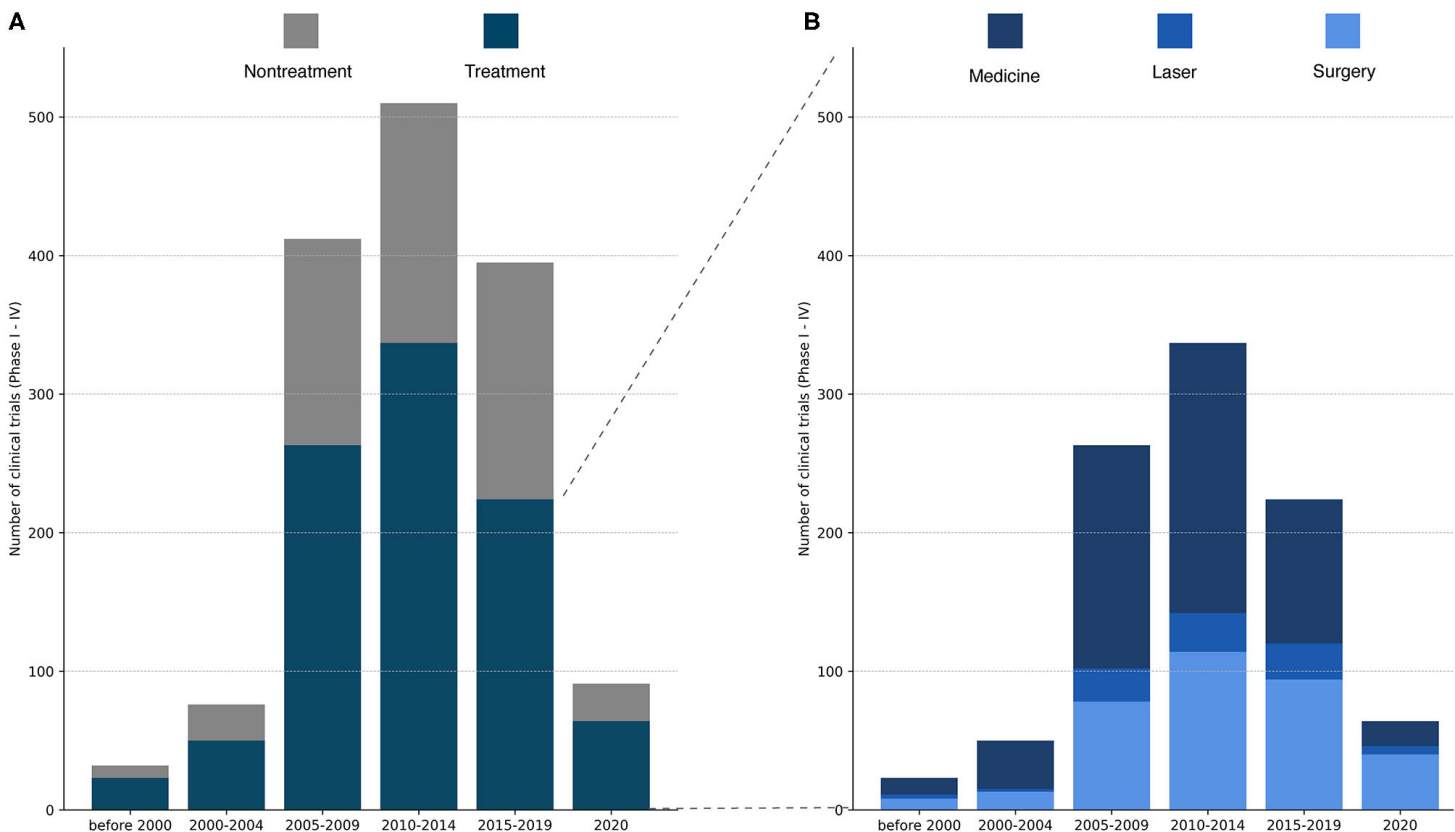
**(A)** Total number of registered glaucoma clinical trials (Phase I–IV) over time. Each study was categorized as treatment or non-treatment. The number of clinical trials increased dramatically from 2000–2004 to 2005–2009, peaking in 2010–2014 and declining in 2025–2019. Of important note, the last column contains only trials from 2020, while the other columns cover a period of at least 4 years. **(B)** Total number of registered clinical trials in the treatment group divided into medical treatment, laser treatment or surgical treatment. Medical treatments constitute the majority of the assessed glaucoma treatments except from 2020, where surgery was the most frequently evaluated treatment in glaucoma trials.

A total of 1,537 trials were identified. One-thousand five hundred and twenty-three trials were identified in the ClinicalTrials database, and in addition, 14 trials were identified in the Australian Clinical Database. The majority of all trials evaluated glaucoma treatment (*n* = 971, 63%, Figure 1A). Within the treatment group, trials of medical treatment dominated, covering a total of 55% (Figure 1B; Table 1). Medical trials accounted for the majority of the trials for a long time. From 2015 to 2019, the proportion of trials evaluating surgery increased, and by 2020, the balance shifted, and trials investigating surgical treatment dominated by 63% of the total numbers of trials. Clinical trials with laser treatment accounted for a small proportion of all trials. The percentage of laser trials has been slightly increasing over time and ranges from 4% in 2000–2004 to 12% in 2015–2019.

**Table 1:** Total number of registered glaucoma clinical trials listed for surgery, laser and medical treatment with total number of trials (*n*, %).

**Year**	**Surgery**	**Laser**	**Medicine**
Before 2000	8 (33%)	3 (13%)	13 (54%)
2000–2004	13 (25%)	2 (4%)	36 (71%)
2005–2009	78 (30%)	24 (9%)	161 (61%)
2010–2014	114 (34%)	27 (8%)	197 (58%)
2015–2019	94 (42%)	26 (12%)	104 (46%)
2020	40 (63%)	6 (9%)	18 (28%)
Total	347 (36%)	88 (9%)	529 (55%)

*In total 347 (36%) trials examined surgery, 88 (9%) examined laser treatment, and 529 (55%) examined medical treatments. In 2020, surgery was the most frequently examined treatment of glaucoma trials, while prior medical treatment accounted for the majority of trials. Laser treatment consistently covered 4–13% of glaucoma trials*.

Most studies evaluated the IOP-lowering effect as primary efficacy outcome (Table 2). Overall, 92% of the trials registered in the two databases evaluated IOP, ranging from 69% (before 2000) to 95% (2010–2014). An increasing number of trials evaluating neuroprotective strategies have been registered, reaching 13% in 2015–2019, but overall, this subgroup of studies accounts for only a small proportion of the total number of trials (5%) over time. Evaluation of vascular targets or compounds represented 11% of the studies in 2020–2021 with an overall representation of 3% over time.

**Table 2:** Trials evaluating glaucoma medical treatment categorized by treatment strategy: IOP lowering, vascular or neuroprotective.

**Year**	**IOP lowering**	**Vascular**	**Neuroprotective**
Before 2000	9 (69%)	2 (15%)	2 (15%)
2000–2004	33 (92%)	2 (6%)	1 (3%)
2005–2009	152 (94%)	4 (2%)	5 (3%)
2010–2014	187 (95%)	4 (2%)	6 (3%)
2015–2019	89 (86%)	1 (1%)	14 (13%)
2020	15 (79%)	2 (11%)	1 (5%)
Total	485 (92%)	15 (3%)	29 (5%)

*IOP-lowering agents cover the vast majority of clinical trials in glaucoma. A total of 485 (92%) evaluated IOP-lowering treatments, 15 (3%) evaluated drugs acting on microvascular blood flow, and 29 (5%) trials examined neuroprotective treatments*.

**Figure 2:**
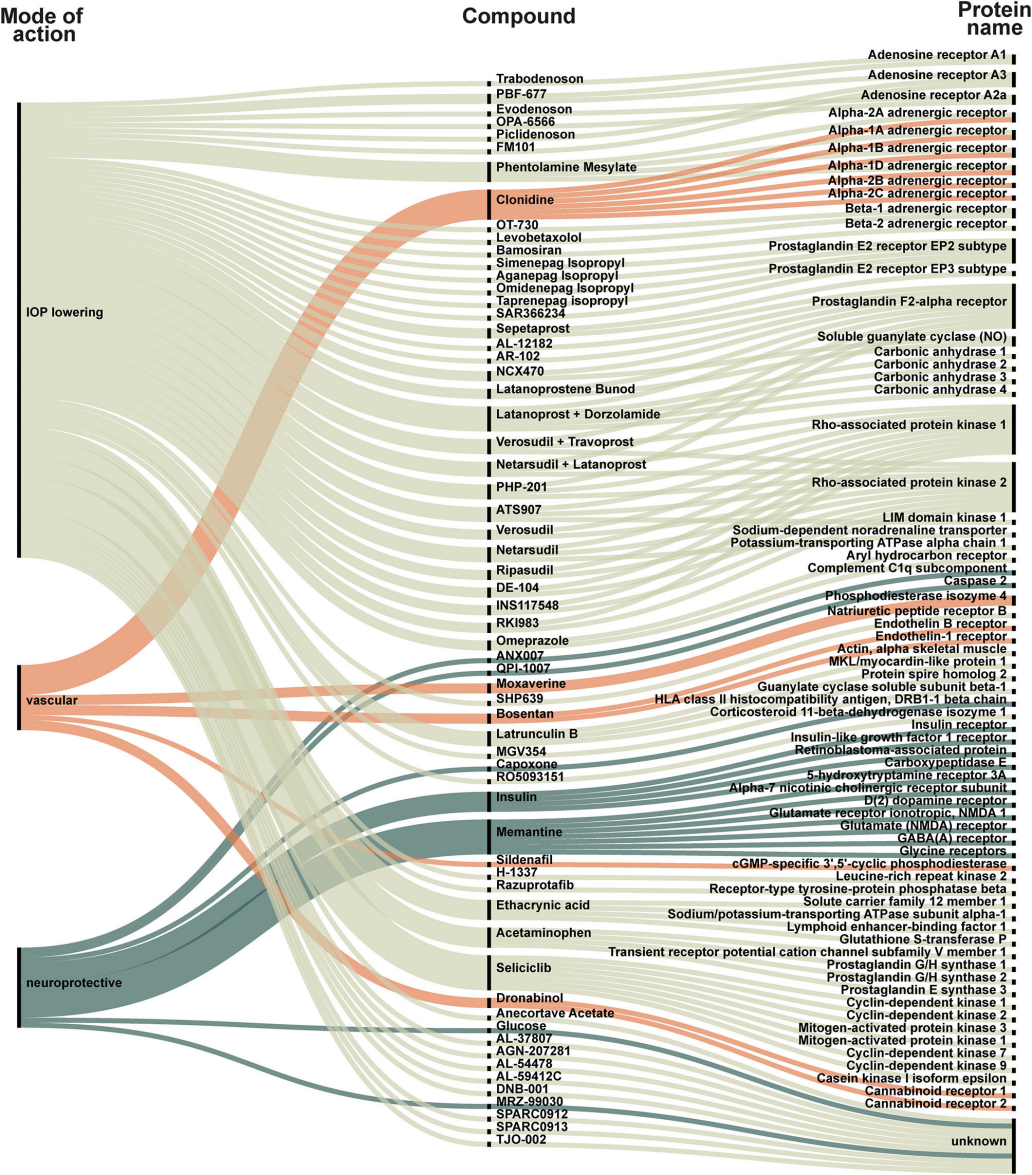
New compounds represented in all included trials. Each compound is categorized as IOP-lowering (light green), vascular (orange) or neuroprotective (dark green). The compound is linked to its target. The targets are listed to the right and visualize how some compounds act on several targets. For example: (1) Trabodenoson is an IOP-lowering compound that targets adenosine receptor A1; (2) Clonidine is a vascular compound targeting six different alpha-adrenergic receptors (1A, 1B, 1D, 2A, 2B, and 2C); (3) Insulin is a neuroprotective compound that acts on insulin receptor, insulin-like growth factor 1 receptor, retinoblastoma-associated protein, and carboxypeptidase E.

**Figure 3:**
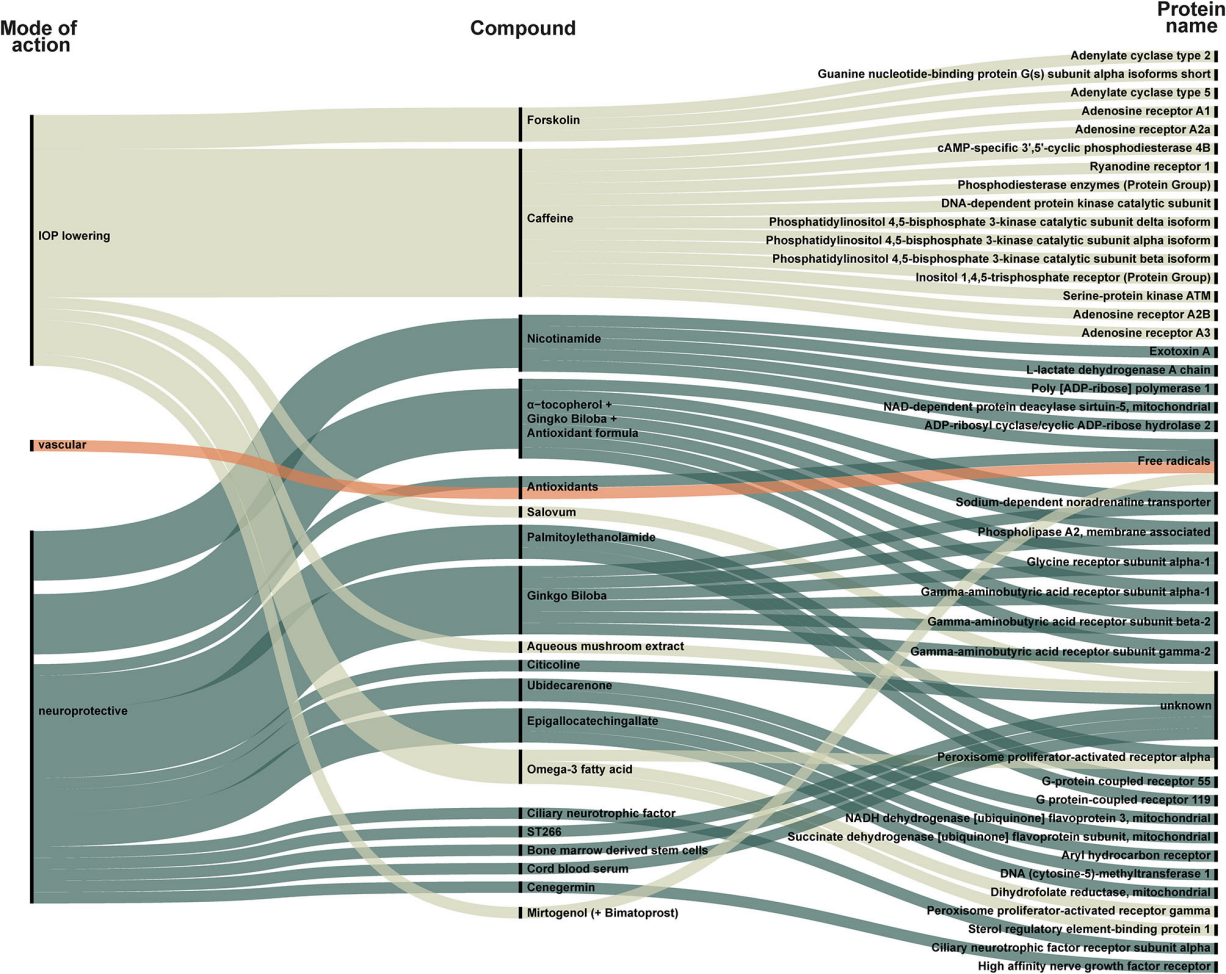
New compounds classified as supplements, stimulants, or transplant identified in all included trials. Each compound is categorized as IOP-lowering (light green), vascular (orange), or neuroprotective (dark green). The compound is linked to its target. The targets are listed to the right and visualize how some compounds act on several targets. For example, forskolin is an IOP-lowering compound that targets adenylate cyclase type 2, G(s) subunit alpha-isoforms short, and adenylate cyclase type 5. Only one group of vascular compounds, namely antioxidants, that act on free radicals, is represented.

We identified 88 new compounds including supplements and a few compounds that investigated a secondary IOP-lowering effect of non-glaucoma treatment (Figures 2, 3). The compounds were sorted by category, phase, drug name and target. Most drug candidates are named, while others are listed with codes used by individual pharmaceutical companies. 11 compounds are labeled “unknown” as the mode of action is unknown.

Due to differences in the regulation of dietary supplements and drugs, we present dietary supplements separately in Figure 3. Figure 3 also includes trials evaluating growth factors and stem cell therapy. A total of 20 compounds were categorized as supplement, stimulants, or transplants (Figure 3).

**Figure 4:**
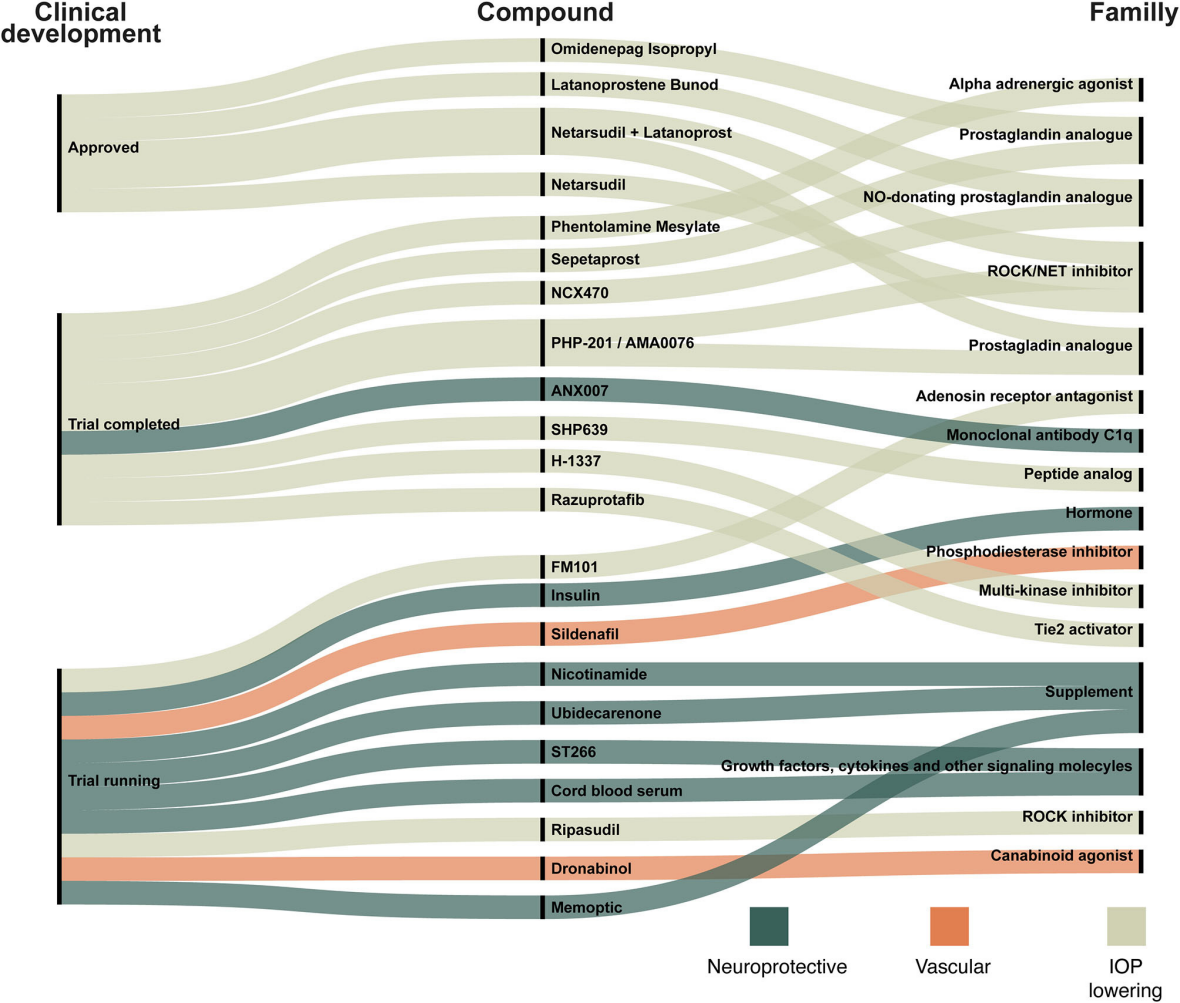
New compounds in ongoing trials, trials completed within 2 years and compounds resulting in recently approved glaucoma treatment drugs. The compounds are linked to the target family listed on the right site. The three colors indicate whether a compound is categorized as neuroprotective (dark green), vascular (orange), or IOP-lowering (light green). For example: (1) Omidenepag Isopropyl, a prostaglandin analog, has been approved and works by lowering IOP; (2) ANX007 is a neuroprotective monoclonal antibody c1q and trials with this compound have been completed; (3) Sildenafil is a phosphodiesterase inhibitor that acts on the microvascular blood flow and trials are still ongoing.

Figure 4 shows recently approved glaucoma treatments (approved within 5 years, *n* = 4) along with pipeline compounds defined as candidates seen in clinical trials with study start date of the study within the last 4 years (*n* = 16).

**Figure 5:**
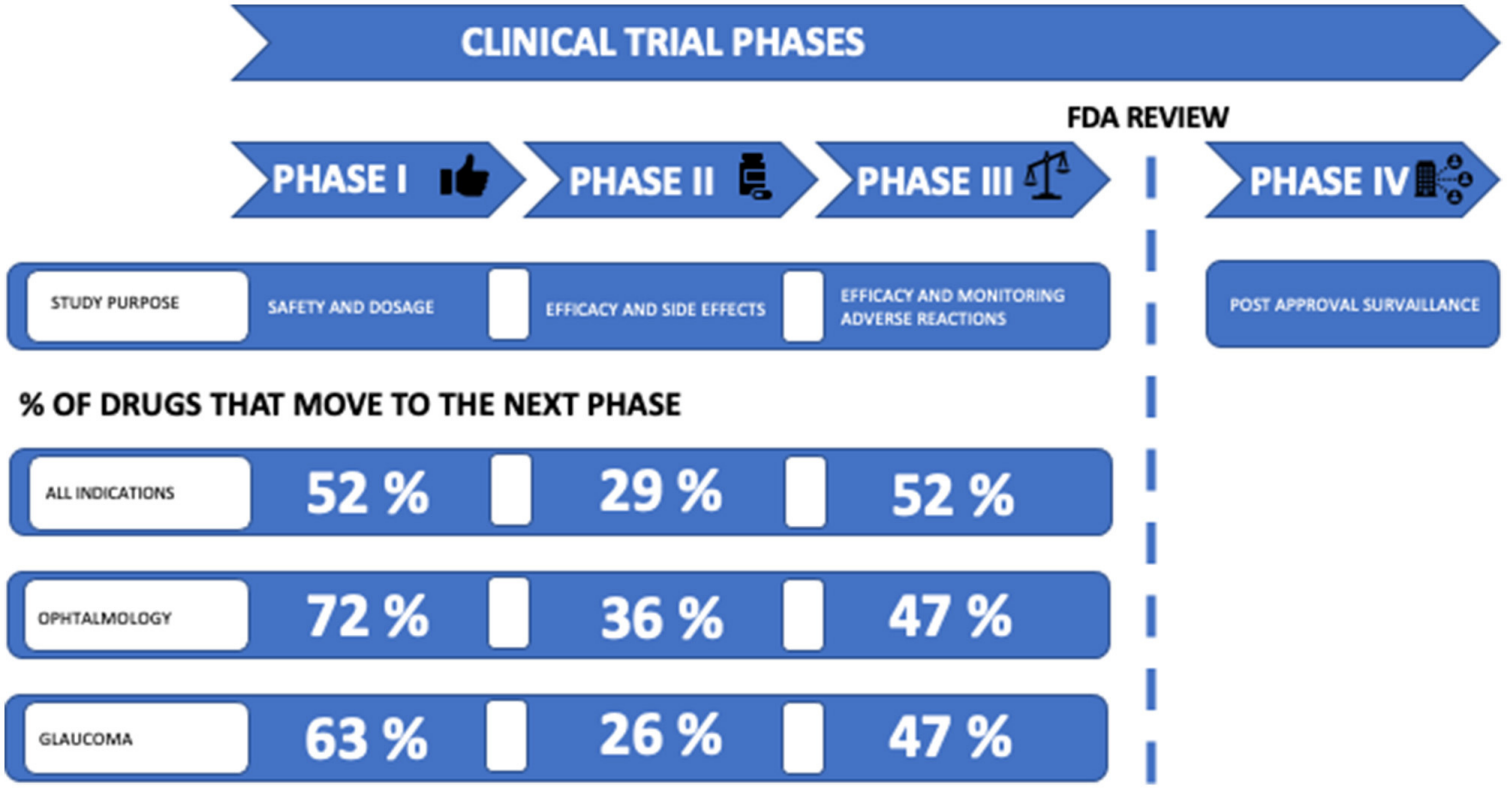
Clinical development success rates. Clinical trial phases are linked to study purpose; phase I studies investigate safety and dosage, phase II studies investigate efficacy and side effects, phase III studies investigate efficacy and monitoring of adverse events, and phase IV studies investigate post-approval surveillance. Before entering phase IV, an FDA review is required. The percentage of drugs that proceed to next phase is listed. Success rates from Table 3 are listed as “Glaucoma” and compared with success rates for ophthalmology and for all indications in the period 2011–2021 in general. Source: BIO (Biotechnology, Innovation, Organization) (47).

The success rates for the transition of the drug development phase were 63% in phase I, 26% in phase II and 47% in phase III (Table 3; Figure 5). The number of compounds that succeed decreases during each phase.

**Table 3:** Success rates for new compounds in glaucoma clinical trials 2000–2020.

**Phase**	**Success per phase**	**Compounds per phase, total (*n*)**	**Success rate (%)**
Phase I	24	38	63
Phase II	12	46	26
Phase III	9	19	47

*Success per phase represents the number of compounds that reach next phase. Compounds per phase represents total number of agents in each phase. Success rate represents the percentage of compounds that succeed to the next phase. 24/38 (63%) compounds succeeded from phase I, 12/46 (26%) compounds succeeded from phase II, and 9/19 (47%) compounds succeeded from phase III*.

## Discussion

### Pipeline Drugs and Tendencies in Glaucoma Clinical Trials

The number of clinical trials evaluating both non-treatment and treatment of glaucoma has increased significantly over the last 30 years. There is a tendency for relatively fewer clinical trials of medical treatment compared to surgical and laser treatment. However, medical treatment is still an essential part of glaucoma treatment, and several trials are assessing traditional and new approaches to medical treatment. We found 105 different drug targets, but these targets compromise only a small proportion of the possible drug targets of glaucoma, as there are more than 2,700 possible targets associated with glaucoma (48). We identified 88 new compounds, 22 of which are of particular interest, as they are either recently approved for glaucoma treatment or possible future candidates (Figure 4). The compounds listed in Figure 4 are discussed below.

### Prostaglandin Analogs and NO-Donating Prostaglandin Analogs

Prostaglandin analogs (PGAs) reduce IOP by targeting prostaglandin F receptor (PTGFR) and prostaglandin E receptor (PTGER 1-4) to increase the outflow of aqueous humor primarily through the uveoscleral pathway, but significant effects have also been reported on trabecular outflow facility (27, 49, 50). Several PGAs have been evaluated in clinical trials over the past decades to find new PGAs with improved tolerability and therapeutic benefits. Omidenepag Isopropyl (DE-117) is a selective PTGER2 agonist currently under development for the treatment of glaucoma and ocular hypertension (OHT) (51–53). Based on results from phase III trials, it received approval in Japan in September 2019 for this indication (53, 54). In February 2021, Santen and Ube Industries announced that the FDA had accepted the New Drug Application for Omidenepag Isopropyl (55).

Sepetaprost is a prodrug that is hydrolyzed by esterase to its active metabolite. It was developed as a dual agonist of PTGFR and PTGER3, in contrast to the classical PGAs, which function primarily through PTGFR (56, 57). With this new dual-mechanism approach, IOP reduction may be improved. A phase IIb dose-finding study was completed in 2018 [NCT03216902]. Enrolment has just begun for a multicenter phase II trial to evaluate efficacy and safety of Sepetaprost [NCT04742283].

Nitric oxide (NO), which donates PGAs, has been evaluated in clinical trials since the late 2000s. A Phase IIb dose-finding study was completed in 2018 [NCT03216902]. The release of NO results in further IOP-lowering effect through increased trabecular meshwork outflow by cAMP-mediated relaxation of trabecular meshwork cells (58, 59). Non-IOP related physiological functions of NO may also be important in terms of glaucoma pathophysiology and treatment. Since NO is an important regulator of vascular tone, it has been suggested that NO helps maintain adequate blood supply to the optic nerve (59, 60). A possible neuroprotective effect of NO has been evaluated in preclinical studies, but needs to be investigated further (59). The first NO-donating PGA was approved by FDA in 2017, a NO-donating latanoprost derivative Vyzulta. Another compound, NCX 470, is composed of the prostamide Bimatoprost with an NO-donating moiety. NCX 470 recently advanced to phase III clinical development [NCT04445519].

### Adenosine Receptor Agonists

There are four subtypes of adenosine receptors, described as A_1_, A_2A_, and A_2B_ and A_3_ receptor. Adenosine receptors are widely distributed throughout the body including the eye. Adenosine receptors are numerous in ocular tissue, such as the ciliary body, the trabecular meshwork, the sclera, and the retina (61). We identified nine clinical trials that evaluated compounds acting on an adenosine receptor, but to date none of the compounds have succeeded in progressing to FDA approval (62–64). A single phase I/IIA trials is currently underway [NCT04585100]. FM101 is an oral tablet formulation of an A_3_ receptor modulator that has been shown to be safe in preclinical studies (65).

### Small Molecule Inhibitor of VE-PTP

A phase II clinical trial evaluating Razuprotafib/AKB-9778 was completed in November 2020 [NCT04405245]. AKB-9778 binds to and inhibits vascular endothelial protein tyrosine phosphatase (VE-PTP), an important negative regulator of Tie2. In mice, VE-PTP was expressed by Schlemm's canal endothelium. Topical ocular administration of AKB-9778 increased Tie2 activation, enhanced SC filtration area, and increased outflow facility, resulting in reduced IOP. The effects appear to be mediated by both eNOS activation and Rho kinase pathway inhibition (66).

### Multikinase Inhibitor

H-1337 is a multikinase inhibitor proposed to stimulate the drainage of aqueous humor from the main outflow tract *via* the trabecular meshwork and the Schlemms canal. The mechanism of action of H-1337 has been explained in two pathways, LRRK2 (leucine-rich repeat kinase 2) inhibition as the main pathway and ROCK inhibition as the secondary pathway. LRRK2 is a type of serine/threonine kinase that acts to control the polymerization of intracellular microtubules. When LRRK2 is inhibited, the microtubules that make up the cytoskeleton of trabecular meshwork cells in the eye depolymerize and change the structure. The results of phase I/IIa trial completed in September 2018 [NCT03452033] showed that IOP was reduced vs. placebo and H-1337 was well tolerated (67, 68).

### C-type Natriurectic Peptide Analog

TAK-639/SHP-639 is a topical, 9-amino acid, synthetic, C-type natriuretic peptide analog in phase I development for the treatment of patients with OHT and POAG. The mechanism for lowering IOP is relaxation of the trabecular meshwork *via* activation of the type B natriuretic peptide receptor (NPR-B). TAK-630 has shown potential as an ocular hypotensive agent in preclinical studies (69, 70). Data from the phase I clinical trial report marginal reduction in IOP from baseline values, but only at the highest dose group and at the most frequent dosing regimen (71).

### α-antagonist

Phentolamine has been studied in preclinical studies with promising IOP-lowering effect (72). In 2019, phentolamine was evaluated in a clinical phase II trial for multiple ocular indications. Data were recently published (73) and it was found that phentolamine did not significantly reduce IOP in patients with glaucoma or OHT. There was a tendency for a greater decrease in patients with lower IOP at baseline (73). The authors suggest combination therapy with topical prostaglandins as a possible way for further development of phentolamine in POAG (73).

### ROCK Inhibitors

Among the few new compounds introduced since latanoprost in 1996 are the rho-associated protein kinase (ROCK) inhibitors. Ripasudil 0.4% ophthalmic solution was approved in Japan in September 2014 (74), and netarsudil 0.02% in 2017 by FDA US (75). We found 36 clinical trials investigating the effect of ROCK inhibitors, either as monotherapy (*n* = 5) or combination therapy with prostaglandin analogs (*n* = 31). As ROCK inhibitors were first approved in Japan, the numbers of clinical trials are likely to be higher than 36, as we did not conduct a search in clinicaltrials.jp.

ROCK is a serine/threonine protein kinase and is divided into ROCK1 and ROCK2. ROCK1and ROCK2 are activated by a GTP-binding protein named Rho A. When ROCK1 or ROCK2 are phosphorylated and activated by Rho A, they act on several intracellular molecules such as myosin light chain, LIM-kinase, calponin and ERM. This range of functions contributes to the different features of ROCK inhibitors, as they act directly on the trabecular meshwork and Schlemm's canal by modulating cell adhesion, cell motility, proliferation, and cell differentiation (76). The mentioned actions lead to a reduction in IOP. In addition, several studies suggest that ROCK inhibitors act on multiple other parameters, and both neuroprotection and beneficial effects on retinal blood flow may increase its anti-glaucomatous effects (76). Overall, ROCK inhibitors are well tolerated, with conjunctival hyperemia as the most common adverse effect of treatment. As ROCK inhibitors act on different targets compared to traditional IOP-lowering agents, combining ROCK-inhibitors with other IOP-lowering drugs may be an additive, which may be a future strategy when monotherapy is not sufficient to achieve disease control.

### Vascular Compounds

Interest in finding treatment strategies other than lowering IOPs has increased in recent decades. As our data suggest, research into vascular compounds is still limited with 15 trials conducted over time. In 2019, a phase III trial investigating phosphodiesterase (PDE) inhibitors effect on blood circulation in the retina and the choroid was initiated [NCT04052269]. PDE is an enzyme subgrouped into 12 isoenzymes. The isoenzymes are differently distributed in various tissues in the body. For example, PDE5 is found in platelets and vascular smooth muscle cells in the corpus cavernosum. PDE5 inhibitors are used primarily to treat of erectile dysfunction and work by increasing blood circulation in the corpus cavernosum. PDE5 is also present in the choroidal and retinal vessels (77). In the above-mentioned study, the drug was dispensed as an oral pill and not as a topical administration. The aim of the study was to investigate whether it is possible to detect changes in blood flow in the retina and choroid assessed by OCT scans.

In 2020, a phase II trial was launched to investigate retinal blood flow [NCT04596826]. The trial focuses on dronabinol, a synthetic Tetrahydrocannabinol (THC) agent. THC binds to two cannabinoid G protein coupled receptors: CB_1_ and CB_2_. CB_1_ receptors are distributed in neurons and a variety of peripheral organs and tissues, such as endocrine glands, leucocytes, spleen, heart, reproductive system, urinary tract, and gastrointestinal tracts. CB_2_ is distributed in the immune system. Thus, binding to CB_1_ affects the psyche and circulation, while CB_2_ does not (78). The study mentioned above investigated whether THC affects the retinal hemodynamic after oral administration evaluated by OCT, retinal vessel diameter, retinal oxygen saturation, and retinal blood velocity among others.

Data from the two trials examining PDE5 and THC have not yet been published. The small number of trials evaluating compounds acting on microvascular blood may be an indicator of insufficient methods to evaluate the effect of a particular compound on ocular blood flow. Suitable endpoints are needed for clinical trials to determine if modification of ocular blood flow is an effective therapeutic target for glaucoma.

### Neuroprotective Compounds

Neuroprotection in glaucoma can be addressed from several starting points. Among many deprivation of neurotrophic factors, excitotoxicity and oxidative stress are some examples (79). The compounds that have been investigated in clinical trials for neuroprotection in glaucoma can be categorized accordingly.

Neurotrophic factors (NFs) are soluble polypeptides with several functions in the nervous system. They are important for the survival, maintenance, and regeneration of neuronal cells (80). Depletion of various neurotrophic factors has been associated with specific disease pathology.

Brain-derived neurotrophic factor (BDNF), which is known to regulate neuronal survival and function in the central nervous system, plays an important role in the treatment of neurodegenerative diseases and has been associated with Parkinson's, Alzheimer's, and Huntington's diseases (80).

ST266 is a novel biologic drug candidate made by a method of culturing amnion epithelial cells harvested from the placenta after birth (81). It contains biologically active proteins and other factors that promote wound healing and preservation of retinal ganglion cells (RGC). A phase I trial is currently running [NCT03901781] (81). Similarly, a study investigating eye drops prepared from umbilical cord blood serum containing growth factors is registered in clinicaltrial.gov, but recruitment status is unknown and last updated in 2018 [NCT03609125].

Citicoline (cytidine 5-diphos-phocholine) is an FDA-approved supplement. Citicoline is an endogenous molecule that participates in the synthesis of membrane proteins. It has been shown to be beneficial in ischemic stroke, traumatic brain injury, Parkinson's disease, Alzheimer's disease, and cerebrovascular diseases (19). In animal models, citicoline has an anti-apoptotic effect on RGC by decreasing glutamate excitotoxicity and oxidative stress (19, 79, 82). Clinical trials do not show consistent results. Parisi et al. (83) showed improvement on retinal function and neural conduction along the visual pathways. Marino et al. (84) found improvement in contrast sensitivity and quality of life, but there were no significant effects on the visual field. A new trial is recruiting patients to evaluate the effect of Memoptic; citicoline in combination with ginkgo biloba, magnesium, vitamin B5 and zinc on visual field performance [NCT04499157].

Nicotinamide, also known as niacinamide, is a water-soluble form of vitamin B3 and is an FDA-approved dietary supplement. Nicotinamide is the precursor of nicotinamide adenine dinucleotide (NAD), which is a coenzyme in several cellular processes, including energy metabolism and DNA repair. Aging causes a decrease in NAD levels leading to metabolic and mitochondrial dysfunction, leaving RGC more prone to cell death. Animal models show that nicotinamide prevents RGC death during IOP elevation. Improvement of mitochondrial function also protects RGCs (85, 86). A clinical trial is currently underway to show whether nicotinamide is beneficial in humans for visual field-testing performance [NCT03797469]. Early results from NCT03797469 have shown a promising neuroprotective potential of nicotinamide. Thus, Hui et al. (87) have shown improved inner retinal function measured by photopic negative response after 3 months treatment with nicotinamide.

Oxidative stress is also a likely contributing factor in the pathogenesis of glaucoma. Studies have shown that antioxidants such as Coenzyme Q10, alpha-lipoic acid, superoxide dismutase, ginkgo biloba leaf extract, and bilberry leaf extract decrease RGC loss in rat models of glaucoma (88).

Another contributing factor to glaucomatous loss of RGC is low-grade inflammation, also called neuroinflammation (89–91). ANX007 is an investigational monoclonal antibody antigen binding fragment (Fab) for the treatment of patients with complement-mediated neurodegenerative ophthalmic diseases. ANX007 completed phase I and has progressed into phase II trial, however, for geographic atrophy and not for glaucoma [NCT04656561] (92).

Insulin resistance has been associated with neurodegeneration in diseases characterized by dendritic pathology, notably Alzheimer's and Parkinson's disease. Insulin may promote the regeneration of dendrites following traumatic injury (93). A phase I trial using topical insulin for glaucoma patients is recruiting patients [NCT04118920].

Neuroprotection in glaucoma is certainly an important approach to treating glaucoma. However, as Liu and Pang (41) describe, there are the following challenges in the discovery and development of neuroprotective drugs: uncertain mechanisms of pathogenesis, uncertain therapeutic targets, preclinical models yet to be validated, and limitations in clinical detection of disease progression.

A general challenge in ophthalmological research is the choice of primary endpoint. As mentioned earlier, IOP is the only evidence-based treatable risk factor for slowing the worsening of glaucoma. Thus, the primary endpoint of most trials is related to IOP, making it difficult to evaluate potential neuroprotective properties of the drug, as progression of visual field defects or OCT scans are rarely evaluated alongside to IOP control. Using electrophysiology, hereunder photopic negative response (87), as a primary endpoint along with biomarkers to stratify treatment effectiveness can help improve positive outcomes of ongoing and future glaucoma trials.

The ultimate goal of drug development is to introduce a promising new compound with a proven therapeutic effect on the market. It is a milestone when a compound moves from preclinical to clinical phase. However, <10% of the drugs entering clinical trials will be approved by regulatory authorities (94). Consistent with previous studies of success rates for the transition to the next phase of drug development, we found the success rate for phase II to be lower than any other phase (46, 95, 96). In this study, we found that the success rate in phase II was 26% (Table 3; Figure 5). Thomas et al. (47) recently reported a phase II success rate for all indications of 29%. We found that the transition success rates for phase I and phase III were 63 and 47%, respectively. Although glaucoma compounds are more successful in progressing from phase I than reported by Thomas et al. (52%), we found the success rate from phase III to approval to be lower than reported by Thomas et. al. (47) (58%). Thomas et al. (47) report phase transition success rates for major disease areas. In ophthalmology, the phase I, II, and III transition success rates were 72, 36, and 47%, respectively. Thus, the phase transition success rates in clinical glaucoma research appear to be below the general level in ophthalmology.

Gower et al. (97) suggest that personalized medicine could be a way to improve the success rate in ophthalmic research. When randomizing people to clinical trials, biomarkers and underlying disease pathophysiology should be considered. This can potentially improve the sensitivity of analysis by investigating whether a particular subgroup responds better to a current treatment.

Gower et al. argues that the lack of discovering new drugs may be due to the special properties of the eye, such as the immediate dilution of the eye drops caused by the tear film, which results in reduced effect of the drug. To succeed with the new strategies in glaucoma research, it is very important to continue to improve knowledge and understanding of the pathogenesis of glaucoma in order to optimize disease modeling and thereby improve the understanding of potential new targets and compounds with promising opportunities (97). In a review by Gross et al., the authors suggest that biophysical models could provide an effective tool in glaucoma risk assessment and may improve the understanding of the inconsistency in treatment response among patients. The biophysical models and mathematical modeling are still at an early stage, and it is challenging to incorporate, e.g., the circadian curve of IOP into the models (98). Also, genetic prediction models could potentially identify individuals most at risk for disease development and progression (99). As previously mentioned, more than 2,700 potential targets for glaucoma exists, but only a fraction are represented in clinical trials. Drug compounds targeting glaucoma risk genes may be potential therapeutic candidates. New technologies using genetic information could also assist in predicting if a patient likely would benefit from a certain treatment. With future innovative drug development where the above-mentioned aspects of personalized medicine and mathematical modeling are considered, we might see an increasing phase transition success rate in glaucoma clinical trials.

## Conclusion

The number of clinical trials in glaucoma research has increased significantly over the last 30 years. A total of 1,537 trials were identified, with the majority evaluating glaucoma treatment (*n* = 971, 63%). Medical treatments covered 55% of these trials over time with IOP evaluation as the most common primary endpoint. In 2020, clinical trials evaluating surgical treatment dominated with 63%. Eighty-eight new compounds were identified with 22 compounds that are currently in clinical development or recently approved for glaucoma treatment. New PGAs and NO donating PGAs are in late-stage development. Dietary supplements currently being evaluated for neuroprotective effects are nicotinamide, citicoline in combination and Coenzyme Q10. Overall, research into neuroprotection and vascular modalities is still sparse with non-uniform results. However, the early results of the nicotinamide trial give rise to some hope for an IOP-independent treatment for glaucoma. The phase transition success rates were below the level of success rates in ophthalmology, potentially caused by the particular anatomy and physiology of the eye, thus reducing the effectiveness of a drug. A future strategy to improve the success rates in glaucoma and ophthalmological research can be achieved from personalized medicine. This paper contributes to the literature by highlighting the difficulties of finding other treatment strategies than lowering IOP and by showing, that only a fraction of the new drugs reaches the market despite comprehensive research. It underlines the need for further research in the complex pathophysiology of glaucoma and for future innovative drug development.

## Author Contributions

The manuscript has been conceptualized by MK. LS and TT have performed data collection and analysis with advices from MK and AH. AH has designed the figures with input from all authors. LS, TT, and JF have written the manuscript with supervision from MK. All authors have provided feedback and helped shape the data analysis and final manuscript.

## Conflict of Interest

The authors declare that the research was conducted in the absence of any commercial or financial relationships that could be construed as a potential conflict of interest.

## Publisher's Note

All claims expressed in this article are solely those of the authors and do not necessarily represent those of their affiliated organizations, or those of the publisher, the editors and the reviewers. Any product that may be evaluated in this article, or claim that may be made by its manufacturer, is not guaranteed or endorsed by the publisher.
